# ATP-induced Ca^2+^-signalling mechanisms in the regulation of mesenchymal stem cell migration

**DOI:** 10.1007/s00018-017-2545-6

**Published:** 2017-05-22

**Authors:** Lin-Hua Jiang, Fatema Mousawi, Xuebin Yang, Sėbastien Roger

**Affiliations:** 1 School of Biomedical Sciences, Faculty of Biological Sciences, University of Leeds, Woodhouse Lane, Leeds, LS2 9JT UK; 2 Sino-UK Joint Laboratory of Brain Function and Injury, Department of Physiology and Neurobiology, Xinxiang Medical University, Xinxiang, 453003 Henan People’s Republic of China; 3 Inserm UMR1069, Nutrition, Croissance et Cancer, Université François-Rabelais de Tours, 37032 Tours, France; 4 Department of Oral Biology, University of Leeds, WTBB, St James University Hospital, Leeds, LS97TF UK

**Keywords:** Extracellular ATP, Ca^2+^ signalling, P2X receptors, P2Y receptors, Store-operated Ca^2+^ channels, Mesenchymal stem cells, Cell migration

## Abstract

The ability of cells to migrate to the destined tissues or lesions is crucial for physiological processes from tissue morphogenesis, homeostasis and immune responses, and also for stem cell-based regenerative medicines. Cytosolic Ca^2+^ is a primary second messenger in the control and regulation of a wide range of cell functions including cell migration. Extracellular ATP, together with the cognate receptors on the cell surface, ligand-gated ion channel P2X receptors and a subset of G-protein-coupled P2Y receptors, represents common autocrine and/or paracrine Ca^2+^ signalling mechanisms. The P2X receptor ion channels mediate extracellular Ca^2+^ influx, whereas stimulation of the P2Y receptors triggers intracellular Ca^2+^ release from the endoplasmic reticulum (ER), and activation of both type of receptors thus can elevate the cytosolic Ca^2+^ concentration ([Ca^2+^]_c_), albeit with different kinetics and capacity. Reduction in the ER Ca^2+^ level following the P2Y receptor activation can further induce store-operated Ca^2+^ entry as a distinct Ca^2+^ influx pathway that contributes in ATP-induced increase in the [Ca^2+^]_c_. Mesenchymal stem cells (MSC) are a group of multipotent stem cells that grow from adult tissues and hold promising applications in tissue engineering and cell-based therapies treating a great and diverse number of diseases. There is increasing evidence to show constitutive or evoked ATP release from stem cells themselves or mature cells in the close vicinity. In this review, we discuss the mechanisms for ATP release and clearance, the receptors and ion channels participating in ATP-induced Ca^2+^ signalling and the roles of such signalling mechanisms in mediating ATP-induced regulation of MSC migration.

## Introduction

Cell migration from one location to another is fundamental to diverse physiological processes ranging from tissue morphogenesis and homeostasis to wound healing and immune surveillance and also to pathological processes such as cancer cell invasion [1–6]. Cell migration is a complex and highly coordinated process. Adhesive cells often migrate in the so-called mesenchymal mode, in which the migrating cell undergo rear-to-front polarization, protrusion and adhesion formation, and rear retraction. All these major steps in cell migration are orchestrated by numerous scaffold, adaptor and adhesion proteins (e.g., actin, myosin, integrin, paxillin and tensin) in concerted actions that are regulated by various signalling molecules, including protein kinase C (PKC), mitogen-activated protein kinases [MAPK; c-Jun N-terminal kinase (JNK), extracellular signal-regulated kinase (ERK) and p38], Rho GTPase, Rho kinase, and focal adhesion kinase [1, 7–9]. As the ubiquitous second messenger, cytosolic Ca^2+^ plays an important role in regulating many cell functions, including cell migration, in response to diverse physical, chemical and biological clues from the surrounding environments [10–20].

Stem cells are a group of specialized cells resident in several tissues or organs in the body. They are endowed with two unique abilities, namely, self-renewal and differentiation. Embryonic stem cells from the inner cell mass of the pre-implantation blastocyst are pluripotent and give rise to almost every cell type, whereas adult stem cells are multipotent and differentiate to the cell types for the tissue or organ in which they reside and, for this reason, these cells are also referred to tissue-specific stem cells. To date, several types of adult stem cells have been identified. For example, hematopoietic stem or progenitor cells (HSC/HPC) in the bone marrow can give rise to all blood cell types, and the bone marrow transplantation is a hematopoietic stem cell-based therapy for diseases like leukaemia, multiple myeloma and lymphoma [21]. Neural stem or progenitor cells (NSC/NPC) are found in the two major neurogenic niches in the brain, the subventricular zone of the lateral ventricle and the subgranular zone within the dentate gyrus of hippocampus. They have the potential of differentiating to neuron, astrocyte and oligodendrocyte, three major cell types in the nervous system and, therefore, are critical in neurogenesis [22]. Cardiac stem or progenitor cells (CSC/CPC) in the heart can generate myocyte, smooth muscle and endothelial cell [23, 24]. Mesenchymal stem cells or multipotent stromal cells (MSC), present in the connective tissue that surrounds other tissues and organs, exhibit differentiation into multiple cell types, including osteoblast, adipocyte, chondrocyte, and potentially muscle cell, myocyte, neuron and glial cell [25–28]. MSC can be easily isolated from several adult tissues, readily expanded in vitro, and exhibit robust immunomodulatory properties. All these highly desirable attributes make MSC to be a stem cell source in the development of regenerative medicines. Indeed, a huge number of preclinical studies have demonstrated promising therapeutic applications of MSC in tissue engineering and cell-based therapy to repair and replace damaged or lost cells and tissues due to a variety of injury or diseases including autoimmune disorders [25, 27–45]. The migrating or homing ability of stem cells to the destined tissues or lesions is not only crucial for normal tissue morphogenesis, homeostasis and repair, but also for development of stem cell-based regenerative medicines [46–54]. There is accumulating evidence to show the importance of Ca^2+^ signalling mechanisms in the regulation of both embryonic and adult stem cell migration [43, 48, 50, 54–70].

ATP is known as the major cellular energy source present at high concentrations inside every living cell, and thus inevitably appears extracellularly at, and in the close vicinity to, the site of tissue damage or inflammation. The ancient and universal availability of ATP prompts the interesting idea that this molecule likely represents the first extracellular signal and purinergic signalling is the primordial form of cell-to-cell communications in multi-cell organisms [71]. Regardless, it has become clear nowadays that in addition to cytolytic leakage from damaged or dying cells, ATP is released via non-cytolytic mechanisms from many cell types and, once outside the cell, it acts as an autocrine and/or paracrine signalling molecule by elevating the cytosolic Ca^2+^ concentration ([Ca^2+^]_c_) via activating the ionotropic P2X receptors and metabotropic P2Y receptors on the cell surface. There is increasing evidence to suggest that ATP-induced purinergic signalling gives rise to significant effects on stem and progenitor cell proliferation, migration and differentiation under in vitro and in vivo conditions [47, 55, 57, 69, 72–91]. In this short review, we aim to give an overview of ATP-induced Ca^2+^ signalling mechanisms mainly in MSC and briefly on other adult stem and progenitor cells. We start with an introduction of ATP release and clearance, and then discuss the receptors and ion channels participating in ATP-induced Ca^2+^ signalling and the role of such signalling mechanisms in ATP-induced regulation of cell migration.

## ATP release and clearance

An earlier study examining the Ca^2+^ signalling mechanisms responsible for the spontaneous oscillations in the [Ca^2+^]_c_ in NSC/NPC derived from embryonic striatum provided the first clue that stem cells can release ATP [56]. The spontaneous Ca^2+^ oscillations were prevented by treatment with apyrase, an ecto-enzyme that, as discussed further below, catalyzes hydrolysis of extracellular ATP. Such a finding strongly suggests constitutive release of ATP as part of the mechanisms generating spontaneous Ca^2+^ oscillations. Spontaneous Ca^2+^ oscillations were later on documented in a subset of human bone marrow-derived MSC (BM-MSC) [79]. Such spontaneous Ca^2+^ oscillations in human BM-MSC were largely halted in the extracellular solution containing glucose, but remained in the glucose-free solution, upon treatment with hexokinase, an enzyme that uses ATP to phosphorylate glucose [79]. Measurement of the ATP content in the cell culture medium using the luciferin/luciferase assay showed that a significant amount of ATP was present in the medium culturing human BM-MSC but not in the cell-free medium [79]. These findings support that human BM-MSC can constitutively release ATP. A subsequent study, also by measuring the concentrations of ATP in the cell culture medium, provided independent evidence to confirm constitutive release of ATP from human BM-MSC under in vitro culturing conditions [76]. There is evidence that human BM-MSC can also constitutively release β-nicotinamide adenine dinucleotide (β-NAD) [60]. ATP release from human BM-MSC was robustly enhanced by mechanical stimuli, such as fluid flow-induced shear stress [77] or shockwave [78]. Similarly, β-NAD release from human BM-MSC was stimulated in response to fluid flow-induced shear stress [60].

**Fig. 1:**
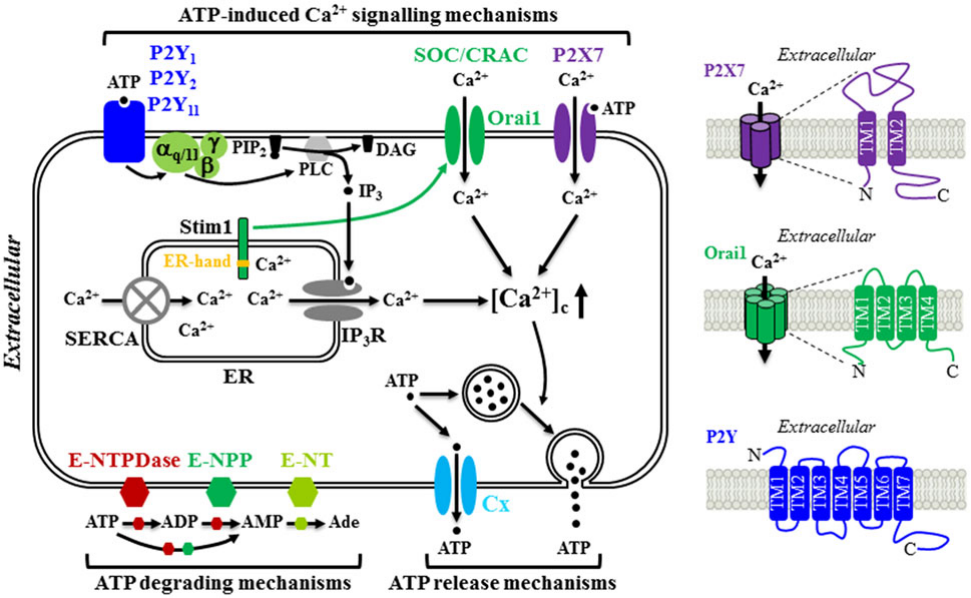
Schematic diagram illustrating the molecular mechanisms for ATP release and hydrolysis, and ATP-induced Ca^2+^ signalling in MSC. MSC release ATP constitutively through connexin (Cx) hemi-channels and in response to mechanical stimuli via vesicular exocytosis. Extracellular ATP are hydrolyzed to ADP and AMP by ecto-nucleoside triphosphate diphosphohydrolases (E-NTPDases) and ecto-nucleotide pyrophosphatase/phosphodiesterase (E-NPP) and further to adenosine (Ade) by ecto-5′-nucelotidase (E-NT). Once outside the cell, ATP acts as an autocrine or paracrine signalling molecule by elevating the cytosolic Ca^2+^ concentrations ([Ca^2+^]_c_) through three molecular mechanisms. ATP can induce activation of the P2X7 receptor ion channel allowing extracellular Ca^2+^ influx. Alternatively, ATP can activate the P2Y_1_, P2Y_2_ and/or P2Y_11_ receptor, leading to sequential activation of G_α,q/11_, phospholipase C (PLC), conversion of membrane lipid phosphatidylinositol 4,5-bisphosphate (PIP_2_) to inositol triphosphate (IP_3_) and diacylglycerol (DAG), activation of the receptor for IP_3_ (IP_3_R) and Ca^2+^ release from the endoplasmic reticulum (ER). Depletion of the ER Ca^2+^, upon activation of the G_α,q/11_-PLC-IP_3_R signalling pathway or blockage of the sarco/endoplasmic reticulum Ca^2+^-ATPase (SERCA) induces extracellular Ca^2+^ entry via the store-operated Ca^2+^ (SOC) or Ca^2+^ release activated Ca^2+^ (CRAC) channel. Stim1 acts as the ER Ca^2+^ sensor via the EF-hand motifs located in the ER lumen (denoted by *yellow strip*) to monitor the ER Ca^2+^ level. Reduction in the ER Ca^2+^ level induces conformal changes in Stim1, leading to its translocation to and trapping at the ER-plasma membrane junction, where it interacts with the Orai1 protein to open the Ca^2+^-permeating channel. Further details and references are described in the text. The structural features of the P2X7 receptor, Orai1 channel and P2Y receptor are illustrated on the right

Constitutive or evoked ATP release has been well documented in many mature cell types, but the underlying mechanisms still remain not fully defined. Studies have shown that exocytosis or secretion of ATP from ATP-containing vesicles in the presynaptic neurons as a co-neurotransmitter or neuromodulator [92, 93] and from ATP-containing lysosome in microglial cells [94] occurs in the central and peripheral nervous systems. Additional molecular mechanisms for ATP release have been proposed, including diffusional movement through a diversity of ion channels and transporters, such as connexin (Cx) or pannexin hemi-channels, cystic fibrosis transmembrane conductance regulator, volume-regulated Cl^−^ channel, P2X7 receptor ion channel, and multidrug resistance transporter [92, 93, 95–98]. Evidence also exists to suggest that the same cells are equipped with multiple ATP release mechanisms and the mechanism used may depend on the situations with which the cells encounter. BM-MSC is one example (Fig. 1). Constitutive ATP release from human BM-MSC cultured in vitro was strongly inhibited by treatment with octanol, palmitoleic acid or 18-α-glycyrrhetinic acid, which are known blockers of the Cx hemi-channels. Such results consistently support a critical role for the Cx hemi-channels in mediating constitutive ATP release [79]. Furthermore, it has been shown that the Cx43 hemi-channel mediates ATP release from pigment epithelium cells [99, 100], whereas the Cx45 hemi-channel serves as the route of ATP release from neural progenitor cells [97]. There is evidence that the Cx43 hemi-channel is functionally expressed in human BM-MSC and plays an important role in mediating constitutive release of β-NAD [60]. However, it remains unclear whether it is involved in constitutive release of ATP. In contrast with constitutive release of ATP, shear stress-induced ATP release from human BM-MSC was insensitive to blockage by 18-α-glycyrrhetinic acid, and instead strongly suppressed by treatment with monensin, an inhibitor of vesicular transport, or treatment with *N*-ethylmalemide to block the fusion of vesicle to the plasma membrane, therefore favouring the notion that ATP is released in response to shear stress via vesicular exocytosis [77]. There is evidence that vesicular release of ATP is Ca^2+^-dependent [57, 94]. However, it is unclear whether shear stress-induced vesicular release of ATP from human BM-MSC is Ca^2+^-dependent and, if it is the case, which Ca^2+^ signalling mechanism is involved. Several recent studies have demonstrated that the newly-discovered Ca^2+^-permeable, mechanosensitive Piezo1 channel plays a critical role in mediating stretch or stress-evoked Ca^2+^ influx and ATP release in urothelial cells [101], red blood cells [102] and endothelial cells [103]. An electrophysiological study has recently reported functional expression of a mechanosensitive stretch-activated Ca^2+^-permeable channel in human MSC derived from desquamated endometrium in menstrual blood, but the molecular identity of this channel has not been established [104]. Therefore, it is interesting to examine whether the Piezo1 channel is expressed in human MSC and plays a role in mechanical stimuli-induced ATP release as well as mechanical stimuli-induced regulation of MSC proliferation and differentiation [77, 78, 105].

In addition to being a physiological signal, extracellular ATP is known as a danger signal because a large quantity of ATP efflux can cause tissue damage and inflammation. Thus, almost all cell types have equipped with some capacity of terminating the action of ATP, particularly protecting against ATP-induced damage, by expressing ecto-nucleotidases that catalyse ATP hydrolysis. Members of the ecto-nucleoside triphosphate diphosphohydrolase (E-NTPDase) family, such as E-NTPDase 1 (ecto-apyrase or CD39) and NTPDase 2 (ecto-ATPase or CD39L1), and the ectonucleotide pyrophosphatase/phosphodiesterase (E-NPP) family represent the major ecto-enzymes that degrade ATP to ADP and AMP, which is further converted to adenosine by ecto-5′-nucelotidase (Fig. 1) [106]. MSC express multiple ecto-nucleotidases, for example, ecto-apyrase in human BM-MSC detected using immunocytochemistry [107] and human MSC derived from gingival tissues in immune-labelling and cell sorting analysis [42], and NPP1 and NPP3 in human BM-MSC shown at the mRNA level using reverse transcription-polymerase chain reaction (RT-PCR) [60]. Consistently, a recent study has shown that the concentrations of ATP released from human BM-MSC into the culture medium in response to shockwave was several-fold higher after suramin was included in the medium to inhibit ecto-nucleotidases and thereby prevent ATP hydrolysis [78].

## Role of P2X receptors in ATP-induced Ca^2+^ signalling

ATP-induced Ca^2+^ responses have been documented in various MSC preparations from human BM, adipose tissues (AT), umbilical cord (UC), periodontal ligament (PDL) and dental pulp (DP) as well as from rat BM and AT (Table 1). ATP-induced increase in the [Ca^2+^]_c_ in the extracellular Ca^2+^-containing solutions was often biphasic, comprising an initial transient component and a sustained component, as shown in human BM-MSC [50, 108] and human DP-MSC [109]. The sustained Ca^2+^ response component was largely lost or significantly attenuated in the extracellular Ca^2+^-free solutions, indicating that Ca^2+^ influx is predominantly responsible for such ATP-induced increase in the [Ca^2+^]_c_.

**Table 1:** Summary of ATP-induced Ca^2+^ signalling mechanisms in MSC

Mechanism	Cell preparations	Supporting evidence	References
P2X	Human BM-MSC	ATP induced an inwardly-rectifying cationic current with a reversal potential of ~0 mV	[76]
	Human AT-MSC	ATP increased the [Ca^2+^]_c_ that was attenuated by NF279, a P2X receptor antagonist	[115]
P2X7	Human BM-MSC	ATP-evoked sustained increase in the [Ca^2+^]_c_ was abolished by KN62, a human P2X7 receptor antagonist	[50]
		BzATP was more potent than ATP in inducing increase in the [Ca^2+^]_c_, large pore formation and membrane blebbing. BzATP-induced effects were inhibited by A-438079, a P2X7 receptor antagonist	[108]
	Human PDL-MSC	BzATP-induced increase in the [Ca^2+^]_c_ and large pore formation were inhibited by oxATP, an irreversible P2X7 receptor inhibitor	[116]
	Human DP-MSC	ATP-induced increase in the [Ca^2+^]_c_ was reduced by AZ11645373, a P2X7 receptor antagonist, and P2X7-specific siRNA	[109]
	Rat BM-MSC	BzATP-induced regulation of differentiation was attenuated by brilliant blue G, a P2X7 receptor antagonist, and P2X7-specific siRNA	[120]
P2Y_1_	Human BM-MSC	Spontaneous Ca^2+^ oscillations were prevented by BzATP, adenosine 3′-phosphate 5′-phosphosulfate or PPADS	[79]
		ATP-induced Ca^2+^-dependent outward K^+^ current was prevented by MRS2179, a P2Y_1_ receptor antagonist	[76]
		ADP and ADPβS were potent in elevating the [Ca^2+^]_c_	[50, 108]
	Human DP-MSC	ATP and ADP induced increases in the [Ca^2+^]_c_, which were reduced by P2Y_1_-specific siRNA	[109]
P2Y_2_	Rat BM-MSC	ATP and UTP, but not ADP and UDP, increased the [Ca^2+^]_c_ in extracellular Ca^2+^-containing and Ca^2+^-free solutions	[130]
		UTP-induced regulation of differentiation was prevented by P2Y_2_-specific siRNA	[131]
P2Y_11_	Human BM-MSC	BzATP, an agonist for the P2Y_11_ receptor, induced an transient increase in the [Ca^2+^]_c_ in extracellular Ca^2+^-free solutions	[50]
	Human DP-MSC	ATP-induced increase in the [Ca^2+^]_c_ was reduced by P2Y_11_-specific siRNA	[109]
SOC/CRAC	Human BM-MSC	Store-operated Ca^2+^ entry was induced, using Ca^2+^ add-back, by TG or CPA-induced ER Ca^2+^ depletion. Highly Ca^2+^-selective SOC channel was activated using CPA, or ER Ca^2+^ release upon activation of the mAChR1-G_α,q_-PLC-IP_3_R signalling pathway	[138]
	Human DP-MSC	Store-operated Ca^2+^ entry was induced, using Ca^2+^ add-back, by TG-induced ER Ca^2+^ depletion. TG-induced store-operated Ca^2+^ entry and ATP-induced increase in the [Ca^2+^]_c_ were inhibited by Synta66, a SOC channel blocker	[109]
Orai1/Stim1	Human DP-MSC	TG-induced store-operated Ca^2+^ entry and ATP-induced increase in the [Ca^2+^]_c_ were reduced by Orai1-specific siRNA and Stim1-specific siRNA	[109]

The P2X receptors are ligand-gated Ca^2+^-permeable cation channels that are activated by extracellular ATP and therefore can mediate ATP-induced Ca^2+^ influx. Mammalian cells express seven genes encoding seven P2X receptor subunits, P2X1-P2X7, with a membrane topology consisting of intracellular N-/C-termini, and two α-helical transmembrane segments (TM1 and TM2) connected by a large extracellular domain (Fig. 1) [110–113]. They form homo-trimers or hetero-trimers, in which ATP binding at the inter-subunit interface of the extracellular part induces conformational changes leading to opening of the Ca^2+^-permeating pathway formed by the TM2 segment from each of the three subunits that allows entry of extracellular Ca^2+^ into the cell to elevate the [Ca^2+^]_c_ [114].

Coppi et al. were the first to show by whole-cell patch-clamp current recording that ATP elicited an inwardly-rectifying current with a reversal potential of ~0 mV in a subset of human BM-MSC [76], providing direct evidence to demonstrate the expression of functional P2X receptor. As highlighted in our recent review, there is noticeable discrepancy in the findings reported by previous studies in terms of the P2X receptor expression at the mRNA, protein and functional levels in MSC derived from different tissues and species [26]. Several studies, using Ca^2+^ imaging, investigated the role of the P2X receptors in mediating ATP-induced Ca^2+^ signalling and, particularly aimed to identify the P2X receptor type that participates in such ATP-induced Ca^2+^ signalling, in combination with pharmacological and/or genetic means. Thus, ATP-elicited increase in the [Ca^2+^]_c_ in human AT-MSC was prevented by treatment with suramin, a P2 receptor generic antagonist, and attenuated by NF279 [115]. NF279 is known as the P2X1 receptor antagonist with a nanomolar potency but, at the high concentration (100 µM) used in the study, it can also inhibit several other P2X receptors. It is therefore difficult to conclude whether the P2X1 receptor is involved in ATP-induced Ca^2+^ signalling but, nonetheless, these results are conistent with the expression of functional P2X receptors and contribution in ATP-induced Ca^2+^ signalling. An earlier study showed that chondrogenic differentiation of mouse BM-MSC was suppressed by treatment with 5-BDBD, a P2X4 receptor selective antagonist, leading the author to put forth that the P2X4 receptor is functionally expressed and mediates ATP-induced Ca^2+^ influx in mouse BM-MSC [75]. In a recent study, we show the P2X4 mRNA expression in human DP-MSC using RT-PCR. However, ATP-induced increase in the [Ca^2+^]_c_ was insensitive to blockage by 5-BDBD, failing to support a significant role for the P2X4 receptor in ATP-induced increase in the [Ca^2+^]_c_ [109].

As summarized in Table 1, several independent studies provide consistent evidence to indicate an important role for the P2X7 receptor in ATP-induced Ca^2+^ signalling in human MSC. Expression of the P2X7 receptor was consistently demonstrated at the mRNA and/or protein levels in human BM-MSC [50, 78, 108], AT-MSC [115], PDL-MSC [116] and DP-MSC [109]. The sustained component of ATP-evoked increase in the [Ca^2+^]_c_ in human BM-MSC was almost completely abolished by treatment with KN62 [50], a human P2X7 receptor selective antagonist. It is well-known that 2′,3′-(benzoyl-4-benzoyl)-ATP (BzATP) is more potent than ATP at the P2X7 receptors and that prolonged activation of the P2X7 receptor induces large pore formation and membrane blebbing, which represent the signature characteristics of the P2X7 receptor activation [117–119]. Both ATP and BzATP evoked large pore formation and membrane blebbing in human BM-MSC as well as sustained increase in the [Ca^2+^]_c_ and, in addition, BzATP was more potent than ATP in inducing these responses [108]. Furthermore, BzATP-induced responses were inhibited by treatment with A-438079 [108], a P2X7 receptor selective antagonist. In human PDL-MSC, BzATP was also effective in increasing the [Ca^2+^]_c_ and inducing the large pore formation, both of which were inhibited by treatment with oxidized ATP (oxATP), an irreversible P2X7 receptor inhibitor [116]. In human DP-MSC, ATP (0.3–300 μM) induced concentration-dependent increases in the [Ca^2+^]_c_ with a half-maximum concentration (EC_50_) of approximately 20 μM [109]. ATP-induced Ca^2+^ responses were attenuated by treatment with AZ11645373, a human P2X7 receptor specific antagonist. BzATP also induced concentration-dependent increases in the [Ca^2+^]_c_ in human DP-MSC. The maximal Ca^2+^ response amplitude induced by BzATP was greater than that induced by ATP. Furthermore, BzATP/ATP-induced Ca^2+^ responses were attenuated by treatment with P2X7-specific siRNA [109]. Collectively, these studies have provided strong evidence to support the expression of functional P2X7 receptor and significant contribution in ATP-induced Ca^2+^ signalling in human MSC. Another recent study shows mRNA and protein expression of P2X7 receptor in rat BM-MSC [120]. Moreover, BzATP-induced regulation of adipogenic and osteogenic differentiation was attenuated by treatment with brilliant blue G, a P2X7 receptor selective antagonist, or after siRNA-mediated knockdown of the P2X7 receptor expression. These findings consistently support the expression of functional P2X7 receptor and further demonstrate its important role in the regulation of MSC differentiation [120]. However, it is worth mentioning the study examining the role of the P2X receptors in ATP-induced Ca^2+^ signalling in rat AT-MSC [121]. In this study, the mRNA transcript was detected for the P2X3 and P2X4, but not the P2X7 and any other P2X subunits. ATP (10–1000 μM) also induced concentration-dependent increases in the [Ca^2+^]_c_, but ATP-induced increase in the [Ca^2+^]_c_ was not affected by treatment with AZ10606120 [121], a P2X7 receptor selective antagonist, thus contradicting the notion that the P2X7 receptor mediates ATP-induced increase in the [Ca^2+^]_c_. The exact reason for such discrepancy in terms of the P2X7 receptor expression in rat BM-MSC and AT-MSC preparations remains unclear.

## Role of P2Y receptors in ATP-induced Ca^2+^ signalling

The P2Y receptors are distinguished from the P2X receptors in their structural and pharmacological properties and also the signalling mechanisms they mediate [122]. First of all, the P2Y receptors belong to the G-protein-coupled receptor superfamily with a membrane topology made of seven α-helical membrane-spanning segments, extracellular N-terminus and intracellular C-terminus (Fig. 1) [123]. Secondly, the P2Y receptors are different from the P2X receptors in their sensitivity to extracellular nucleotides [124–128]. There are eight mammalian P2Y receptor types, P2Y_1_, P2Y_2_, P2Y_4_, P2Y_6_, P2Y_11_, P2Y_12_, P2Y_13_ and P2Y_14_. While all the P2X receptors are exclusively activated by ATP, the P2Y receptors are activated by various nucleotides with a highly different potency or sensitivity. For example, the human P2Y receptors have the following agonist profile: P2Y_1_ (ADP > ATP), P2Y_2_ (UTP ≥ ATP), P2Y_4_ (UTP), P2Y_6_ (UDP > UTP), P2Y_11_ (ATP > UTP), P2Y_12_ (ADP > ATP), P2Y_13_ (ADP > ATP) and P2Y_14_ (UDP~UDP-glucose) [125]. The third major difference lies in the signalling mechanisms. The P2Y receptors are coupled with various G_α_ subunits and downstream signalling pathways. More specifically, the P2Y_1_, P2Y_2_, P2Y_4_, P2Y_6_ and P2Y_11_ receptors (or the P2Y_1_-like receptors; [125]) are linked with the G_α,q/11_, and activation of these receptors leads to sequential activation of phospholipase C (PLC) β, generation of inositol triphosphate (IP_3_), activation of IP_3_ receptor (IP_3_R) and IP_3_R-mediated Ca^2+^ release from the endoplasmic reticulum (ER). Therefore, activation of the G_α,q/11_-PLC-IP_3_R signalling pathway can increase the [Ca^2+^]_c_ via triggering Ca^2+^ release from intracellular stores (Fig. 1). Stimulation of the P2Y_11_ receptor can additionally activate adenylyl cyclase (AC) and promote generation of cyclic adenosine monophosphate (cAMP) via association with the G_α,s_. By contrast, the P2Y_12_-P2Y_14_ receptors (or the P2Y_12_-likeeceptors; [125]) are coupled to the G_α,i_ and activation of these receptors inhibits the AC activity and cAMP generation.

Expression of the P2Y receptors has been examined in human BM-MSC, AT-MSC and DP-MSC, and also rat and mouse BM-MSC at the mRNA, protein and/or functional levels. As introduced above, the P2Y_1_, P2Y_2_ and P2Y_11_ receptors represent the major ATP-sensitive human P2Y receptors that are coupled to the G_α,q/11_-PLC-IP_3_R signalling pathway triggering intracellular Ca^2+^ release. Therefore, our discussion below is confined to the studies examining expression of these three receptors and their role in ATP-induced Ca^2+^ signalling. Two recent reviews provide further details regarding expression of the P2Y receptors and function in MSC [26, 129]. As discussed above, human BM-MSC [50, 108] and human DP-MSC [109] showed biphasic Ca^2+^ responses to ATP in extracellular Ca^2+^-containing solutions. The initial transient increase in the [Ca^2+^]_c_ largely remained in extracellular Ca^2+^-free solutions, indicating ATP-induced Ca^2+^ release from intracellular stores. Such observations provide unambiguous evidence to show the expression of ATP-sensitive P2Y receptors that are coupled to the G_α,q/11_-PLC-IP_3_R signalling pathway. An early study by Kawano et al. showed that the spontaneous Ca^2+^ oscillations were prevented in a large proportion of human BM-MSC by treatment with BzATP, adenosine 3′-phosphate 5′-phosphosulfate or pyridoxal phosphate-6-azo (benzene-2,4-disulfonic acid) (PPADS). These observations led to the proposal that activation of the P2Y_1_ receptor is an essential step in an autocrine/paracrine feedback mechanism sustaining the spontaneous Ca^2+^ oscillations, namely, activation of the P2Y_1_ receptor gives rise to sequential activation of the G_α,q/11_-PLC-IP_3_R signaling pathway, IP_3_R-mediated Ca^2+^ release from the ER, and induction of the store-operated Ca^2+^ entry (discussed below in more detail), and an increase in the [Ca^2+^]_c_ triggers ATP release through the Cx hemi-channels [79] (Fig. 1). Such a signalling mechanism was further supported by the findings that the spontaneous Ca^2+^ oscillations were terminated by treatment with U73122, a PLC inhibitor, or 2-aminoethoxydiphenyl borate (2-APB), a cell-permeant IP_3_R blocker (as discussed below, 2-APB is also known to inhibit numerous plasma membrane Ca^2+^-permeable channels including store-operated Ca^2+^ channel), or over-expression of an IP_3_-binding protein to remove free IP_3_. In the above-mentioned patch-clamp recording study, ATP induced a Ca^2+^-depenent outward K^+^ current as well as a P2X receptor-mediated inward current in an overlapping subset of human BM-MSC [76]. The ATP-induced outward K^+^ current was  prevented by treatment with MRS2179, a P2Y_1_ receptor selective antagonist, suggesting the expression of functional P2Y_1_ receptor and also a role for this receptor in mediating ATP-induced Ca^2+^ signalling. Consistently, a subsequent study, using RT-PCR and western blotting, showed the expression of the P2Y_1_ receptor at both mRNA and protein levels [50]. Further supporting evidence for the expression of functional P2Y_1_ receptor in human BM-MSC was provided by the findings that ADP, a P2Y_1_ receptor selective agonist, and ADPβS, a non-hydrolizable ADP analogue, were potent in elevating the [Ca^2+^]_c_ in these cells [50, 108]. Expsoure to ATP also elevated the [Ca^2+^]_c_ in human AT-MSC, and expression of the P2Y_1_ receptor in these cells was detected at both mRNA and protein levels, using RT-PCR and western blotting, respectively [115]. However, it was not clear whether the P2Y_1_ receptor was involved in ATP-induced Ca^2+^ signalling in human AT-MSC [115]. In human DP-MSC, we have recently shown that ADP as well as ATP induced significant increases in the [Ca^2+^]_c_ and the Ca^2+^ responses induced by both ADP and ATP were reduced by siRNA-mediated knockdown of the P2Y_1_ receptor expression, indicating the expression of functional P2Y_1_ receptor and its contribution in ATP-induced Ca^2+^ signaling [109].

There is evidence for expression of the P2Y_2_ receptor in MSC but the expression of this receptor appears dependent on the tissue and species from which MSC were prepared. An early study demonstrated the mRNA and protein expression of P2Y_2_ receptor in rat BM-MSC [130]. In addition, increases in the [Ca^2+^]_c_ were induced by ATP and UTP, but not ADP and UDP, in both extracellular Ca^2+^-containing and Ca^2+^-free solutions. Such an agonist profile is most consistent with the expression of functional P2Y_2_ receptor in rat BM-MSC [130], which is supported by a recent study that shows that UTP-induced regulation of differentiation was prevented by treatment with P2Y_2_-specific siRNA [131]. However, the P2Y_2_ mRNA expression was at a very low level in human DP-MSC from a 9 year old donor and almost undetectable in cells from two adult donors at the ages of 21 and 32, suggesting that the P2Y_2_ receptor is unlikely to play a major role in ATP-induced Ca^2+^ signalling, at least in human DP-MSC [109].

Strong evidence supports the expression of functional P2Y_11_ receptor in human MSC. In human BM-MSC, β-NAD induced a biphasic increase in the [Ca^2+^]_c_ in the extracellular Ca^2+^-containing solutions, and both the transient and sustained components were prohibited by treatment with NF157, a P2Y_11_ receptor selective antagonist, or siRNA-mediated genetic depletion of the P2Y_11_ receptor expression [132]. It should be pointed out that β-NAD-induced increase in the [Ca^2+^]_c_ is mediated by the P2Y_11_-G_α,s_-AC-cAMP signalling pathway that leads to extracellular Ca^2+^ influx and intracellular Ca^2+^ release mediated by the L-type voltage-gated Ca^2+^ channel in the plasma membrane and the ryanodine receptor in the ER, respectively [132]. Consistently with the expression of P2Y_11_ receptor in human BM-MSC, BzATP, an agonist preferentially activating the P2Y_11_ receptor among the P2Y receptors, induced transient increase in the [Ca^2+^]_c_ in the extracellular Ca^2+^-free solutions, or in the extracellular Ca^2+^-containing solutions in the presence of the P2X7 receptor inhibitor KN62 [50]. In human DP-MSC, there was abundant mRNA expression of the P2Y_11_ receptor. Knockdown with siRNA of the P2Y_11_ receptor expression reduced ATP-induced increase in the [Ca^2+^]_c_, supporting a significant role for the P2Y_11_ receptor in mediating ATP-induced Ca^2+^ signalling [109].

In summary, despite with some discrepancy, studies have accumulated evidence to support functional expression of the P2Y_1_, P2Y_2_ and P2Y_11_ receptors and their contribution in ATP-induced Ca^2+^ signaling (Table 1).

## Store-operated Ca^2+^ entry in ATP-induced Ca^2+^ signalling

Depletion or reduction of the ER Ca^2+^ level, which can be triggered by ATP or numerous other extracellular signals via activation of their cognate G-protein-coupled receptors that are coupled to the G_α,q/11_-PLC-IP_3_R signalling pathway, can further evoke extracellular Ca^2+^ influx, which is commonly called the store-operated Ca^2+^ entry through the store-operated Ca^2+^ (SOC) channels or Ca^2+^ release-activated Ca^2+^ (CRAC) channels [133–135]. An increasing number of studies have shown the store-operated Ca^2+^ entry in mature cells and also in stem and progenitor cells as part of ATP-induced Ca^2+^ signalling mechanism following activation of the P2Y receptors coupled to the G_α,q/11_-PLCβ-IP_3_R signalling pathway [64, 68, 80, 81, 84, 97, 100, 136]. Two distinct proteins, Stim1 and Orai1, have been identified to be critical in mediating the store-operated Ca^2+^ entry through the CRAC channel [133, 134]. Stim1 is a single membrane-spanning protein with the extended N- and C-termini residing in the ER lumen and the cytosol, respectively, and serves as the Ca^2+^ sensor via the N-terminal Ca^2+^-binding EF-hand motifs to monitor the Ca^2+^ level in the ER (Fig. 1). Orai1 protein comprises intracellular N-/C-termini and four α-helical transmembrane segments, and forms a hexameric complex with the first α-helical transmembrane segment from each of the six subunits constituting the Ca^2+^-permeating pathway (Fig. 1) [137]. According to the diffusion-trap model for the store-operated Ca^2+^ entry, Stim1 undergo conformational changes, upon depletion of the ER Ca^2+^, that facilitate its translocation to and trapping at the ER-plasma membrane junction, where Stim1 binds to and thereby gates the Orai1 channel to open [135]. Additional homologue proteins, Orai2, Orai3 and Stim2, have been discovered but their role in mediating the store-operated Ca^2+^ entry remains less well-defined [133, 134].

It has been proposed that the store-operated Ca^2+^ entry contributes to ATP-induced Ca^2+^ signalling in MSC (Table 1). As mentioned above, Kawano et al. showed the store-operated Ca^2+^ entry as an essential part of the autocrine/paracrine feedback mechanism generating spontaneous Ca^2+^ oscillations in human BM-MSC, triggered by ATP-induced activation of the P2Y_1_ receptor and G_α,q/11_-PLCβ-IP_3_R signalling pathway and ensuing IP_3_R-mediated Ca^2+^ release [79, 138]. In an even earlier study, this group showed store-operated Ca^2+^ entry, using the Ca^2+^ add-back protocols that are widely used to record Ca^2+^ influx in the extracellular Ca^2+^-containing solutions in cells that are prior treated in the extracellular Ca^2+^-free solutions with thapsigargin (TG) or cyclopiazonic acid (CPA), inhibitors that specifically block the sarco/endoplasmic Ca^2+^-ATPase (SERCA) and thereby deplete Ca^2+^ in the ER (Fig. 1) [138]. In addition, they also performed patch-clamp recording to demonstrate functional expression of a highly Ca^2+^-selective SOC channel that was activated following treatment with CPA, or acetylcholine (ACh) [138] via activation of the muscarinic ACh receptor 1 (mAChR1) that is coupled to the G_α,q/11_-PLC-IP_3_R signalling pathway and Ca^2+^ release from the ER [62]. However, the molecular identity of the channel mediating the store-operated Ca^2+^ entry in human BM-MSC still remains elusive. In human DP-MSC, ATP induced intracellular Ca^2+^ release in the extracellular Ca^2+^-free solutions, leading to massive Ca^2+^ influx upon adding Ca^2+^ back to the extracellular solutions. We further validated functional expression of the SOC channels, using the Ca^2+^ add-back protocols and TG. Treatment with titrated concentration of 2-APB, or Synta66, a SOC channel specific blocker, reduced TG-induced activation of the SOC channel. Such treatment also attenuated ATP-induced increase in the [Ca^2+^]_c_ in the extracellular Ca^2+^-containing solutions, providing strong evidence to show the store-operated Ca^2+^ entry to be important part of ATP-induced Ca^2+^ signalling mechanism. Furthermore, we have documented the mRNA transcripts for Orai1, Stim1 and Stim2 in human DP-MSC by RT-PCR. Knockdown using siRNA of the expression of Orai1 or Stim1, but Stim2, reduced TG-induced store-operated Ca^2+^ entry and also ATP-induced increase in the [Ca^2+^]_c_ in the extracellular Ca^2+^-containing solutions [109]. These results provide the first evidence to show that the Orai1/Stim1 CRAC channel plays an important role in mediating store-operated Ca^2+^ entry as part of ATP-induced Ca^2+^ signalling mechanism in human DP-MSC (Fig. 1).

## ATP-induced Ca^2+^ signalling mechanisms in the regulation of cell migration

Studies have shown that extracellular ATP induces or stimulates the migration of mature cells, for example, epithelial cells [139, 140] and microglial cells [94]. In particular, extracellular ATP strongly regulates the migrating capacity of cancer cells, which is a critical determinant of cancer invasion or metastasis giving rise to the high casualty [141], and the P2X7 [142–145] and P2Y_2_ receptors [86, 146–151] have been shown to play a critical role in mediating such ATP-induced regulation of cancer cell migration. There is accumulating evidence that extracellular ATP influences stem cell and progenitor cell migration [47, 48, 55, 57, 58, 87, 88] and genetic and/or pharmacological manipulations or disease-associated alterations of the P2X receptors, P2Y receptors and/or SOC channels and associated Ca^2+^ signalling mechanism give rise considerable effects on stem and progenitor cell migration [48, 55, 63–65, 91, 152].

Evidence is also emerging to support that extracellular ATP regulates human MSC migration under in vitro conditions and also their homing capability in vivo [50, 60, 109]. Ferrari et al. were first to demonstrate, using the trans-well migration assay, that application of exogenous ATP in the culture medium in the upper chamber increased human BM-MSC migration [50]. Addition of ATP in the culture medium in the lower chamber as a chemotactic signal resulted in no effect on the cell migration, but enhanced the chemotaxis in response to chemokine CXCL-12. However, as shown in a separate study, addition of ATP in the lower chamber accelerated human BM-MSC migration [60]. In the same study, prior treatment with ATP also stimulated cell migration [60]. Furthermore, addition of β-NAD in the upper or lower chamber enhanced cell migration [60]. The increase in cell migration induced by β-NAD was abolished by treatment with NF157, a P2Y_11_ receptor selective antagonist, and also by treatment with 2′,3′-dideoxyadenosine, an AC inhibitor [60]. These results clearly support critical involvement of the P2Y_11_-G_α,s_-AC-cAMP signalling pathway in stimulation of cell migration by β-NAD. In a recent study, we have shown using time-lapse imaging in combination with the scratch-induced wound healing assay that ATP facilitated human DP-MSC migration [109]. Similar ATP-induced stimulatory effect on human DP-MSC migration was also observed using the trans-well assay [109]. ATP-induced increase in human DP-MSC migration was completely abolished by PPADS, but not affected by CGS15943, a generic inhibitor of adenosine receptors, suggesting that ATP-induced stimulation of cell migration is predominantly mediated by activation of the P2 receptors rather than activation of the adenosine receptors by adenosine, the major by-product of ATP hydrolysis (Fig. 1) [109]. Consistently, ATP-induced increase in human DP-MSC migration was attenuated by treatment with AZ11645373 to inhibit the P2X7 receptor, and also by siRNA-mediated knockdown of the expression of the P2X7, P2Y_1_ or P2Y_11_ receptor. Such ATP-induced increase in cell migration was also suppressed by treatment with 2-APB at a concentration that preferentially inhibits the store-operated Ca^2+^ entry and, more specifically, by siRNA-mediated reduction of the expression of Orai1, Stim1 or both [109]. Taken together, these results provide evidence to show that the major ATP-induced Ca^2+^ signalling mechanisms discussed above, namely, the P2X7, P2Y_1_ and P2Y_11_ receptors and the Orai1/Stim1 channel, participate in ATP-induced stimulation of human DP-MSC migration (Fig. 1).

It is worth making a special note of the study by Ferrari et al. that showed that pretreatment with ATP significantly improved the homing ability of human BMS-MSC after they were planted into immunocompromised mice [50]. Such a finding is therapeutically interesting as it provide the first proof of concept that the in vivo migrating or homing capacity of MSC can be purposely fine-tuned by in vitro priming MSC with ATP.

## Concluding remarks and perspectives

Recent studies have made, as discussed above, significant advances in understanding the molecular mechanisms underlying ATP-induced Ca^2+^ signalling in human MSC. Evidence has emerged to show an important role for such Ca^2+^ signalling mechanisms in extracellular ATP-induced regulation of MSC migration, but more studies are clearly required to provide a more detailed or mechanistic insight. As introduced above, cell migrates in a complex but well-orchestrated process that is often described in three major steps, establishment of a rear-to-front polarity, protrusion and formation of focal adhesions at the front or leading edge, and retraction from the rear edge [1]. There is evidence that Ca^2+^ signalling regulates polarization [69, 70] and adhesion formation [19]. However, it remains unknown which of these steps in MSC migration is regulated by the above-discussed ATP-induced Ca^2+^ signalling. As briefly mentioned above, it is well established that PKC, MAPK and other Ca^2+^-dependent signalling proteins are important regulators of the cytoskeletal proteins coordinating cell migration [1, 7–9]. Studies have shown that Ca^2+^ activates or modulates these signalling molecules in MSC. For example, internal Ca^2+^ release triggered by activation of the G_α,q_-PLC-IP_3_R signalling pathway activates the PKC-ERK1/2 signalling pathway in ACh-induced rat BM-MSC migration [62]. There is also evidence that P2X7 receptor activation in MSC leads to activation of the ERK1/2 and JNK signalling pathways in ATP-induced down-regulation of adipogenic differentiation [120] or the p38 signalling pathway in the up-regulation of osteogenic differentiation induced by shockwave or extracellular ATP [78]. Further studies are required to examine whether such Ca^2+^-dependent signalling pathways are involved in ATP-induced regulation of MSC migration. Finally, the cell migration or the homing capacity of in vitro expanded MSC cultures to the lesion sites is limited but critical for development of regenerative medicines, particularly cell-based therapy. Evidently, more in vivo studies are required to examine whether the improved understanding of the Ca^2+^ signalling mechanisms underlying ATP-induced regulation of cell migration can be harnessed to improve the low or poor homing capacity of MSC and thereby the efficacy of promising applications of MSC-based tissue engineering and therapies.
